# Preclinical Evaluation of mTORC1 Inhibition via Rapamycin in Machine-Perfused Rodent Livers

**DOI:** 10.21203/rs.3.rs-7340727/v1

**Published:** 2025-10-10

**Authors:** Christopher Taveras, Alissa Cutrone, Emmanuella Ajenu, Madeeha Hassan, Alban Longchamp, Shannon Tessier, Korkut Uygun, Heidi Yeh

**Affiliations:** Massachusetts General Hospital; Massachusetts General Hospital; Massachusetts General Hospital; Massachusetts General Hospital; Massachusetts General Hospital; Massachusetts General Hospital; Massachusetts General Hospital; Massachusetts General Hospital

**Keywords:** Rapamycin, Warm Ischemia, Ischemia Reperfusion Injury, Liver Preservation, Normothermic Machine Perfusion, mTORC1 inhibition

## Abstract

Normothermic machine perfusion (NMP) enables real-time liver viability assessment and therapeutic intervention during preservation. Rapamycin, an mTORC1 inhibitor known to induce autophagy and promote cellular resilience, represents a promising candidate for mitigating ischemic injury during NMP. We developed a rat model of warm ischemia followed by NMP to evaluate whether rapamycin improves liver graft quality. Livers were subjected to 90 minutes of warm ischemia and then perfused for three hours with or without 50nM rapamycin. While no significant differences were observed in perfusate oxygen consumption, lactate clearance, or transaminase release, histological analysis revealed that rapamycin-treated livers had improved cellular architecture and reduced necrosis compared to untreated controls. These findings suggest that rapamycin may confer structural protection independent of immediate metabolic changes. This model provides a platform to further study targeted therapies during NMP and supports the potential role of mTORC1 modulation in enhancing liver graft preservation.

## Introduction

Normothermic machine perfusion (NMP) has emerged as a transformative technique in liver transplantation, offering improved graft assessment, preservation, and recovery compared to traditional cold storage methods [1–3]. During NMP, livers are maintained at physiological temperatures, allowing metabolic activity and viability assessment, as well as providing a unique opportunity for therapeutic interventions directly targeting organ viability and function [2, 4, 5].

A potential intervention during NMP is the mechanistic target of rapamycin complex 1 (mTORC1), a critical regulator of cellular metabolism, growth, and survival. mTORC1 integrates nutrient availability, energy status, and growth factor to control processes such as protein synthesis, lipid metabolism, and autophagy, all vital to hepatic function and regeneration [6]. In hepatic physiology, mTORC1 is integral to maintaining liver homeostasis by regulating hepatocyte proliferation and controlling adaptive responses to metabolic stress [6, 7]. mTORC1 inhibits autophagy by sequestering Unc-51 like autophagy activating kinase 1 (ULK1) through phosphorylation, preventing its role in autophagosome formation [8]. Consequently, dysregulated mTORC1 signaling has been implicated in liver pathologies ranging from steatosis and fibrosis to impaired regenerative capacity, further emphasizing its significance as a therapeutic target during liver preservation [7, 9].

Rapamycin, a potent and selective inhibitor of mTORC1, has attracted considerable attention due to its capacity to modulate these pathways and thus activate autophagy [10], allowing the organ to repair itself. Rapamycin functions by first interacting with the intracellular protein FKBP12, forming a complex that subsequently associates with mTORC1, effectively suppressing its kinase activity [11]. Rapamycin (generic name sirolimus) received FDA approval in 1999 as an immunosuppressant and has since been utilized for various clinical applications, including the prevention of kidney transplant rejection, management of lymphangioleiomyomatosis, and treatment of malignant perivascular epithelioid cell tumors [12–15]. Rapamycin’s therapeutic relevance extends beyond traditional clinical applications and is being explored in emerging research for aging, neurological disorders, organ transplantation, and graft-versus-host disease [16–22].

Early findings suggest that rapamycin during NMP may have a therapeutic effect on discarded human grafts, however inherent heterogeneity between discarded grafts such as age, ischemic time, and previous health conditions make it difficult to draw definite conclusions [23]. These findings strongly suggest that rapamycin administration during NMP could significantly enhance liver graft viability, positioning mTORC1 modulation as a promising avenue for improving transplantation outcomes.

We herein describe the first controlled study in a rodent liver model that looks at the administration of rapamycin in for mitigation of warm ischemia damage. We hypothesized that the addition of rapamycin to NMP will improve liver functionality throughout NMP following warm ischemia damage. After ischemic time, the livers were perfused with a blood-based perfusate similar to current NMP protocols used clinically, with the addition of Rapamycin, and results were compared to untreated controls.

## Methods

### Liver Procurement and Warm Ischemia

Male Lewis rats (250–300 g, 8–12 weeks old, Charles River Laboratories, Boston MA, USA) were socially housed in controlled, standard conditions (12-hour light/day cycle, 12C, 30–70% humidity, pathogen-free HEPA filtered ventilated cages, mixed paper/cellulose bedding) as previously described [24]. All rats had unfettered access to sterile water and chow, as in accordance with National Research Council Guidelines [24]. All rats were cared for by the Massachusetts General Hospital (MGH) Center for Comparative Medicine (CCM). The experimental protocol was approved by the Institutional Care and Use Committee (IACUC) of MGH (Protocol #2011N000111), and all experiments were performed in accordance with established ARRIVE guidelines. Livers were procured as previously described [25]. Donor rats were anaesthetized under 3–5% isoflurane. The abdomen was opened via a transverse abdominal incision. Ligaments connecting the superior and inferior portions of the liver were dissected and the portal vein was exposed. The gastric and splenic branches of the portal vein, as well as the hepatic artery were ligated using 6–0 silk. The bile duct was partially dissected, cannulated using 24g tubing, and secured with 6–0 silk. The inferior vena cava was heparinized with 1 U/g using a 30-gauge insulin syringe. Following 5 min of heparin circulation, the portal vein was cannulated with a 16g cannula, followed by transection of the IVC. The cannula was connected to 16g tubing attached to a 50 mL syringe containing 1 mL heparin in 60 mL saline. The portal vein was hand flushed at 10 mL/min for 4 min, after which the remaining connective tissue was dissected, and the liver was removed from the body cavity. Following removal, the liver was flushed with the remaining 20 mL saline and then weighed. The liver was then submerged in saline and placed in an airtight bag in 37°C incubator for 90 minutes to incur warm ischemia injury.

### Machine Perfusion and Rapamycin Administration

Normothermic machine perfusion was carried out as previously described [25]. Perfusate circulation was carried out using a roller pump system (Masterflex L/S, Vernon Hills, IL) with two separate sets of tubing delivering perfusate into and out of the 500 mL perfusion reservoir. The system was consistently kept at a temperature of 37°C via a water bath (PolyScience, Niles, Illinois, USA) continuously pumping heated water through the double-jacketed perfusion system components (Radnoti, Covina, CA, USA). Perfusate oxygen concentration was maintained within a close range of 500 mmHg using a 95 % O_2_/5 % CO_2_ gas cylinder (Airgas, Radnor, PA, USA). For the experiment group, before the liver was connected to the system, a 50nM dose of rapamycin suspended in DMSO (Thermo Fisher Scientific, Waltham, MA) was added directly to the basin (n=4). In controls, an equal volume of DMSO was added (n=3). System pressure was zeroed, the liver was placed in the tissue bath and connected to the system. The flow rate was brought from 5 mL/min to 30 mL/min gradually, maintaining a maximum portal pressure of 5 mmHg. Outflow samples were collected every 30 min from the Inferior Vena Cava (IVC), while inflow samples were collected from a port placed above the cannula perfusing the portal vein. The liver was weighed upon the end of perfusion to determine weight change. Biopsies of the left lateral lobe (LLL) were formalin-fixed for histological analysis.

### Perfusion Parameters Assessment

Every 30 min, inflow and outflow perfusate samples were analyzed using a Siemens Rapidpoint 500 (Siemens, Munich, Germany). Perfusion parameters were analyzed to determine liver functionality during perfusion (pH, O_2_ consumption, lactate clearance) as previously described [25]. Additionally, hourly outflow samples were analyzed for hepatic injury markers (AST and ALT enzymes) using a Piccolo Xpress (Abaxis, Union City, CA, USA). Portal resistance was determined using pressure readings taken every half hour, and defined as pressure divided by flow, normalized to weight (g). Oxygen consumption was defined as portal inflow pO_2_ minus IVC outflow pO_2_. Weight change was defined as final weight minus initial weight divided by initial weight.

### Histology and Scoring

Liver tissue samples were sectioned and stained with hematoxylin-eosin (H&E). Slides were imaged under a 20× objective using a NanoZoomer HT whole-slide scanner (Hamamatsu Photonics, Hamamatsu City, Japan). There were 3 slides analyzed for each condition, and from each slide, five representative regions were selected by an unblinded author, randomly sorted, and distributed to three blinded authors for histological scoring. Tissues were evaluated across four categories—steatosis, inflammation, necrosis, and fibrosis—each graded on a 0 to 3 scale [26–29]. Scores across the four categories were averaged followed by statistical analysis.

### Statistical analysis and illustrations

Data are presented as mean ± SD, and differences are considered significant when p < 0.05. Statistical analysis was performed with Prism 8 software Version 9.1.2 (Graphpad Software, San Diego, CA, USA, graphpad.com). Two-way analysis of variance (ANOVA) was performed to compare time-dependent perfusion data, followed by Tukey’s post-hoc test to examine statistical difference. All illustrations were created with Biorender (Toronto, ON, Canada).

## Results

### Liver Viability following Rapamycin Administration:

**Figure 2:**
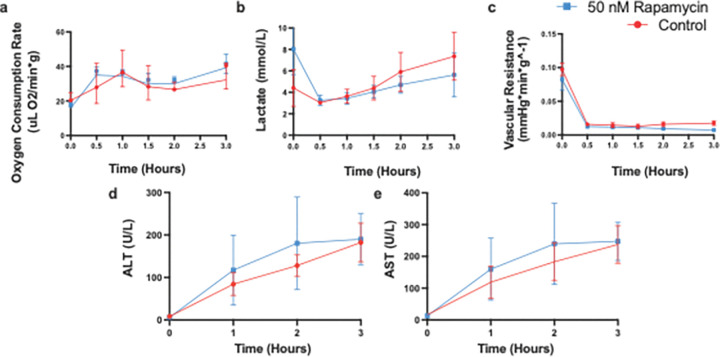
Rapamycin Post-Ischemia Does Not Alter Perfusion Parameters During 3-Hour NMP. No significant change with (a) oxygen consumption, (b) lactate clearance, or (c) portal vein vascular resistance. Additionally, no change was seen in functionality related enzymes (d) ALT and (e) AST.

Throughout the 3-hour NMP period, inflow and outflow samples were collected to assess oxygen consumption and lactate levels (Fig. 2a–b). Oxygen consumption for all groups initially measured around 20 μL O_2_/min*g but increased to 35–40 μL O_2_/min*g within the first 30 minutes, remaining stable for the remainder of the perfusion (Fig. 2a). No significant differences were observed between the rapamycin-treated and control groups. Lactate levels, initially variable (2.5–10 mmol/L) due to residual lactate in the blood perfusate, stabilized at 3.5–4.5 mmol/L within 30 minutes (Fig. 2b). While lactate concentrations gradually increased over the perfusion, the 50nM rapamycin group appeared to have a slower rate of increase, though the slope regression was not statistically significant. Portal vein flow rates and pressures were continuously monitored throughout the perfusion to calculate vascular resistance. These measurements revealed no significant differences in vascular resistance between the control and rapamycin-treated groups (Fig. 2c). Additionally, aspartate aminotransferase (AST) and alanine aminotransferase (ALT) levels were measured to assess hepatocellular injury, with no significant differences observed between the groups (Fig. 2d–e).

### Liver Pathology following Rapamycin Administration:

**Figure 3:**
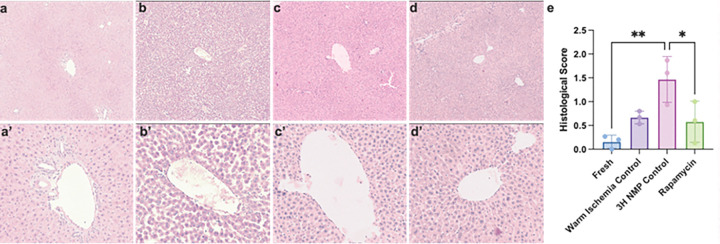
Histology reveals notable differences in Rapamycin Treated Livers. Representative H&E-stained histology of liver tissue at 5x (top) and 20x (bottom). (a) Fresh control liver, (b) Warm Ischemic Liver, (c) Liver perfused for 3 hours via NMP without rapamycin, and (d) Liver preserved for 3 hours via NMP with rapamycin treatment. (e) Data are presented as mean ± standard deviation. Statistical analysis was performed using one-way ANOVA with Tukey’s multiple comparisons test. **p = 0.0063; *p = 0.0485.

The histological analysis of liver tissue revealed notable differences in cellular architecture across experimental groups (Fig. 3a–d). Fresh control livers (Fig. 3a) displayed uniform hepatocytes with clearly defined cell boundaries, intact cytoplasm, and centrally located nuclei, characteristic of healthy liver tissue. In contrast, livers subjected to 90 minutes of warm ischemia (Fig. 3b) exhibited slight tissue damage, including inflammation, cellular swelling, and necrosis. Livers preserved for 3 hours via NMP without rapamycin (Fig. 3c) demonstrated substantial loss of cellular boundaries, extensive necrosis, and disrupted hepatocyte organization, indicating ongoing cellular injury despite perfusion support. However, livers preserved for 3 hours via NMP with 50nM rapamycin (Fig. 3d) displayed improved histological integrity, including better-defined cell boundaries and reduced necrosis, suggesting that rapamycin may have mitigated some of the cellular damage associated with prolonged perfusion. Together, these histological findings highlight the potential benefits of rapamycin in maintaining hepatocellular structure during extended NMP.

## Discussion

While prior studies have largely investigated rapamycin as a preconditioning agent in systemic in vivo models, our study is among the first to examine its direct application NMP [30–33]. We observed that rapamycin treatment improved histological preservation in livers exposed to warm ischemia during perfusion, with treated grafts showing reduced necrosis and better cellular architecture compared to controls. In contrast, perfusate measurements of oxygen consumption, lactate clearance, and transaminase release remained similar between groups.

These findings are partially consistent with previous rodent liver ischemia-reperfusion studies where rapamycin improved histological appearance and decreased ALT levels following reperfusion [32]. However, most of these studies administered rapamycin prior to ischemia, often hours or days in advance, and in the context of systemic inflammation and immune priming [30, 31, 34]. Our work differs by applying rapamycin directly during ex vivo reperfusion, isolating its effects on the liver without systemic variables. Notably, Jiang et al. (2019) reported improved hepatocyte viability and metabolic recovery when rapamycin was given prior to ischemia-reperfusion injury [32]; in contrast, our study suggests that structural benefits may emerge independently of immediate biochemical recovery when administered during perfusion. This distinction shows the need to better understand the temporal dynamics and compartment-specific roles of mTOR signaling during organ preservation.

We established a rat model that enables the evaluation of rapamycin’s effect during normothermic perfusion following warm ischemic injury. While this system provides a controlled platform to assess histological and biochemical outcomes, the current protocol may be enhanced for sensitive to detect subtle protective effects. Modifications such as extending the perfusion duration or increasing the severity of ischemic injury may enhance the model’s ability to reveal treatment-dependent differences. These adjustments could improve the dynamic range of injury and recovery, allowing for a more rigorous assessment of rapamycin’s therapeutic potential.

This study has several limitations. First, the relatively short duration of NMP may not have allowed sufficient time for rapamycin’s effects on metabolic recovery to manifest, potentially explaining the discordance between histological and biochemical endpoints [35]. Second, while histological analysis revealed improved architecture in treated livers, this assessment is inherently limited by sampling variability and lack of functional follow-up, such as post-reperfusion or transplant outcomes [36]. Third, the study was conducted in a rodent model, which may not fully recapitulate the complexity of human donor livers.

To address these limitations, future studies will extend perfusion durations and incorporate functional assays, including bile production and metabolic flux analysis. Importantly, we are currently conducting ongoing experiments with discarded human livers to evaluate the translational potential of rapamycin during NMP under clinically relevant conditions [23]. These studies will combine biochemical monitoring with quantitative histological scoring and transcriptional profiling, offering a more comprehensive understanding of rapamycin’s impact on graft viability and immune signaling. Ultimately, integrating structural and metabolic endpoints across experimental models and time points will be essential to refine viability criteria and guide therapeutic strategies for rapamycin-enhanced liver preservation.

## Figures and Tables

**Figure 1:**
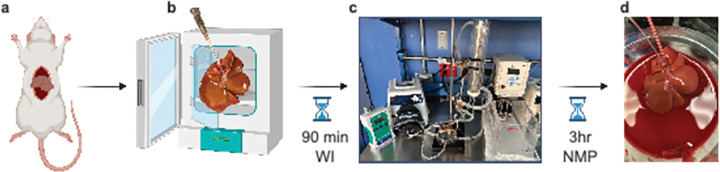
Schematic Representation of Warm Ischemia Induction and Perfusion Protocol. (A) Liver procurement following laparotomy. (B) Incubation in an airtight bag inside of a 37°C incubator for 90 mins to incur warm ischemia damage. (C) Normothermic machine perfusion system with temperature and pressure control. (D) Liver following 3hr NMP with 20% whole rat blood.

## Data Availability

The authors confirm that the primary data supporting the findings of this study are included in the article. Additional information is available from the corresponding author upon reasonable request.
